# Identification of a novel *MIP* frameshift mutation associated with congenital cataract in a Chinese family by whole-exome sequencing and functional analysis

**DOI:** 10.1038/s41433-018-0084-5

**Published:** 2018-04-26

**Authors:** Xigui Long, Yanru Huang, Hu Tan, Zhuo Li, Rui Zhang, Siyuan Linpeng, Weigang Lv, Yingxi Cao, Haoxian Li, Desheng Liang, Lingqian Wu

**Affiliations:** 1 Center for Medical Genetics, School of Life Sciences, Central South University, 110 Xiangya Road, Changsha, 410078 Hunan PR China; 2 Hunan Jiahui Genetics Hospital, 110 Xiangya Road, Changsha, 410078 Hunan PR China

## Abstract

**Purpose:**

To detect the underlying pathogenesis of congenital cataract in a four-generation Chinese family.

**Methods:**

Whole-exome sequencing (WES) of family members (III:4, IV:4, and IV:6) was performed. Sanger sequencing and bioinformatics analysis were subsequently conducted. Full-length WT-*MIP* or K228fs-*MIP* fused to HA markers at the N-terminal was transfected into HeLa cells. Next, quantitative real-time PCR, western blotting and immunofluorescence confocal laser scanning were performed.

**Results:**

The age of onset for nonsyndromic cataracts in male patients was by 1-year old, earlier than for female patients, who exhibited onset at adulthood. A novel c.682_683delAA (p.K228fs230X) mutation in main intrinsic protein (*MIP*) cosegregated with the cataract phenotype. The instability index and unfolded states for truncated MIP were predicted to increase by bioinformatics analysis. The mRNA transcription level of K228fs-*MIP* was reduced compared with that of WT-*MIP*, and K228fs-MIP protein expression was also lower than that of WT-MIP. Immunofluorescence images showed that WT-MIP principally localized to the plasma membrane, whereas the mutant protein was trapped in the cytoplasm.

**Conclusions:**

Our study generated genetic and primary functional evidence for a novel c.682_683delAA mutation in *MIP* that expands the variant spectrum of *MIP* and help us better understand the molecular basis of cataract.

## Introduction

Congenital cataract is a highly clinically and genetically heterogeneous disorder. To date, approximately 36 nonsyndromic congenital cataract-related genes have been identified. The identified genes encode crystalline, cytoskeleton-associated proteins, membrane transport, channel proteins, and some growth and transcription factors.

Although some main intrinsic protein (*MIP*) mutations linked to congenital cataracts have been identified in mice and humans, the heterogeneous phenotypes observed imply an intricate mechanism for *MIP* function. MIP is an intrinsic membrane protein that constitutes over 45% of the total membrane protein in lens fiber cells [1]. As a water channel protein, MIP plays an important role in the transportation of water and small neutral solutes [2–4]. Mutations in *MIP* damage the water homeostasis or permeability of the lens and lead to cataracts in mice [1, 5]. MIP also plays an important role in cell-to-cell adhesion in lens fiber cells [6, 7]. Previous cataract studies showed that most *MIP* mutations decreased protein expression and impaired normal protein localization [2, 8–12]. However, the detailed mechanisms underlying specific mutations remain hidden behind phenotypic heterogeneity, requiring further research.

In this study, whole-exome sequencing (WES) and related functional analysis were performed to detect the underlying pathogenesis of congenital cataract in a four-generation Chinese family.

## Materials and methods

### Patients

**Fig. 1:**
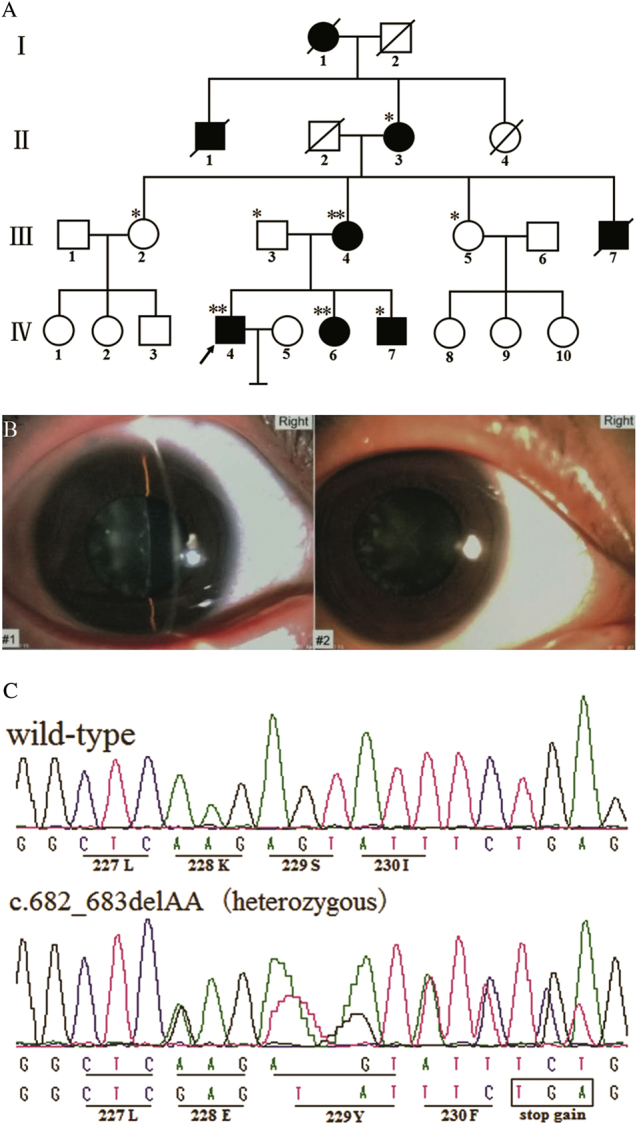
**a** Pedigree of the four-generation Chinese family with congenital cataracts. Squares and circles, respectively, indicate males and females. Empty symbols and filled symbols, respectively, indicate unaffected individuals and affected individuals. The arrow and diagonal lines, respectively, indicate the proband and a deceased individual. Family individuals whose DNA was analyzed with whole-exome sequencing and Sanger sequencing are marked by double asterisks, and individuals analyzed by Sanger sequencing are indicated by a single asterisk. **b** Photograph of the right eye of the patient (IV: 6). Slit-lamp photograph showing the phenotype of punctate cortical opacities. The same phenotype was noted bilaterally. The age of onset for IV:6 was 19 years old. The symptoms were less severe than in the male patients. Other affected family members underwent cataract extractions. **c** DNA sequences of *MIP* in unaffected and affected individuals. A novel heterozygous frameshift mutation (c.682_683delAA, p.K228fs) in *MIP* was detected. The mutation was not detected in unaffected family members or in 100 control samples

A four-generation family living in Hunan Province, China, was recruited, and members of the family were diagnosed with nonsyndromic congenital cataract (Fig. 1a). Clinical and detailed ocular examinations were performed. One hundred normal individuals were recruited as normal controls. This study was consistent with the Tenets of the Declaration of Helsinki and was approved by the ethics committee of the Hunan Jiahui Genetics Hospital. Informed consent was obtained from the subjects.

### Exome capture and sequencing

Using phenol–chloroform extraction, genomic DNA was extracted from peripheral blood cells. WES of affected family members (III: 4, IV:4, and IV:6, Fig. 1a) was performed. Exon-containing fragments were enriched with the xGen® Probes Panel (Integrated DNA Technologies, USA), then sequenced with the HiSeq 2000 platform (Illumina, San Diego, CA) using the paired-end method. Variants identified in three patients were filtered according to the criteria (see supplement-Fig. 1).

### Sanger sequencing

The primers were designed by Primer PREMIER Version 5.0 (Premier Biosoft International, Palo Alto, CA, USA) (sense/anti-sense-1, see supplement-Table 1). The PCR products were sequenced with an ABI 3130 DNA sequencer (Applied Biosystems, Carlsbad, CA, USA). One hundred unrelated normal controls were also analyzed the possible polymorphisms.

### Bioinformatics analysis

See the detailed description in the supplement-text.

#### Human MIP cDNA and plasmid construction

The primers (sense/anti-sense-2, see supplement-Table 1) were used to amplify the coding sequence of wild-type *MIP* (complementary DNA (cDNA) was donated by the Han Lab of Xiamen University, which provides the full-length human cDNA to researchers from non-profit research institutes in China, http://hanlab.xmu.edu.cn/cdna/). The mammalian expression vector pCMV-N-HA containing an N-terminal HA tag (Beyotime, Shanghai, China) was used for plasmid construction. To introduce the mutation into WT-*MIP*, the Mut Express II Fast Mutagenesis Kit V2 (Vazyme Biotech, China) was used according to the manufacturer’s protocol. Site-directed mutagenesis was performed with the primers (sense/anti-sense-3, see supplement-Table 1). The constructs were validated by direct sequencing.

#### Cell culture and transient transfection

Dulbecco’s modified Eagle’s medium (DMEM) supplemented with 10% fetal bovine serum, was used to culture HeLa cells. Wild-type (WT-*MIP*), mutant-*MIP* (K228fs-*MIP*), and control plasmids (empty pCMV-N-HA) with an HA tag were transfected separately using Lipofectamine 2000 (Invitrogen Corporation, Carlsbad, CA, USA) according to the manufacturer’s protocol.

#### Relative mRNA expression

Total RNA was extracted by Trizol (Invitrogen, Carlsbad, CA, USA) from HeLa cells after 48-h transfection. RNA was reverse transcribed into cDNA using the RevertAid First Strand cDNA Synthesis Kit (Thermo Scientific, Carlsbad, CA, USA). Quantitative real-time PCR was performed with a Bio-Rad CFX96 Touch Deep Well Real-Time PCR Detection System (Bio-Rad, Berkeley, CA, USA) using SYBR Premix Ex Taq (Thermo Fisher, Carlsbad, CA, USA) with primers against *MIP* (sense/anti-sense-4, see supplement-Table 1) and β-actin (sense/anti-sense-5, see supplement-Table 1) (https://primerdepot.nci.nih.gov/). Four replicates were analyzed for all of the samples.

#### Protein expression

HeLa cells were lysed in the mixture containing SDS (Sodium Dodecyl Sulfate) buffer and protease inhibitor cocktails (inhibits serine, cysteine, aspartic and metallo proteases) (Sigma-Aldrich, USA) after 48-h transfection and the harvested protein was quantitated using the BCA Protein Assay Kit (Thermo Fisher Scientific, USA) according to the kit instructions. Protein were separated by sodium dodecyl sulfate polyacrylamide gel electrophoresis with 12% acrylamide, then the transferred and blocked protein was incubated with mouse anti-HA monoclonal antibody (catalog numbers: AH158, Beyotime, Shanghai, China) at 1:1000 dilution and monoclonal anti-β-actin IgG (Sigma) at 1:6000 dilution, followed by peroxidase-conjugated affinipure goat anti-mouse IgG (Jackson ImmunoResearch, USA) at a 1:7000 dilution. Enhanced chemiluminescence (Thermo Fisher Scientific, Rockford, USA) was used and then scanned to visualize the specific protein band.

#### Confocal laser scanning immunofluorescence analyses

The cells were fixed with 4% paraformaldehyde for 20 min and permeabilised with 0.2% Triton X-100 for 10 min after 48-h transfection. Then, the samples were incubated with mouse monoclonal anti-HA antibodies (Beyotime, Shanghai, China) and counterstained with 4,6-diamidino-2-phenylindole (DAPI) for 3 min, followed by Cy3 goat anti-mouse IgG (Jackson ImmunoResearch, USA). The cells were analyzed using a Leica confocal microscope (Germany).

## Results

### Patient clinical information

A four-generation Chinese family diagnosed with nonsyndromic congenital cataract was identified (Fig. 1a). The proband (IV: 4) was a 25-year-old male who exhibited bilateral cataracts at 5 months old. Strabismus and nystagmus also existed. His lens was removed. His younger sister (IV: 6, 21 years old, onset at 19 years old, bilateral punctate cortical opacities cataract; Fig. 1b), younger brother (IV: 7, 18 years old, onset at 3 months), mother (III: 4, 42 years old, onset at 30 years), grandmother (II:3, 74 years old, onset at 38 years), uncle (III:7, onset at 9 months, deceased) and two other ancestors (I:1 and II:1, deceased) also showed similar nonsyndromic cataracts and had received cataract surgery. The age of onset in male patients was within the first year of life, earlier than in female patients, who experienced onset in adulthood. Female patients usually showed fewer symptoms. Patients had binocular vision ranging from 0.1 to 0.5.

### Exome sequencing results, variant analysis, and validation

According to the screening step described in the Materials and methods section, 158 variants shared by the three patients were detected in coding regions and adjacent intronic regions. We exhaustively analyzed candidate genes that were previously identified to cause hereditary cataracts.

Some variants, such as frameshift or stop gain mutations, could infect the function were also shown (see supplement-Table 2). By genealogical verification, we identified a heterozygous nucleotide change, c.682_683delAA, in the *MIP* gene (Fig. 1c). Compared with the unaffected family members or the 100 unrelated normal controls, the mutation cosegregated well with the cataract phenotype of the diseased individuals.

### Bioinformatics analysis of K228fs at the protein level

See the detailed description in the supplement-text.

#### The expression of HA-WT-MIP and HA-K228fs-MIP in HeLa cells

**Fig. 2:**
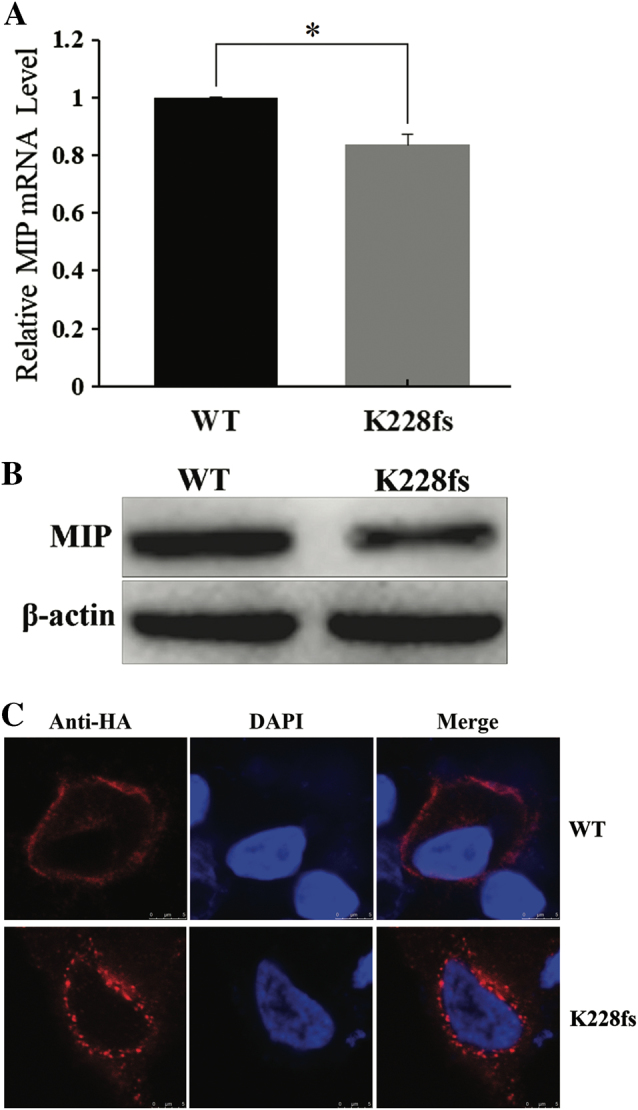
Expression of WT-*MIP* and K228fs-*MIP* in cultured HeLa cells. **a** The mRNA transcription level of K228fs-*MIP* was decreased compared with that of WT-*MIP* according to quantitative real-time PCR (*T*-TEST, two-sided, *P = 0.0052, error bars represent standard deviation, SD). All of the samples were analyzed in four replicates and normalized to median β-actin expression. **b** Western blots were performed as indicated and showed that the protein expression of K228fs-MIP was lower than that of WT-MIP. The expression of β-actin was used as the control. **c** Subcellular localization of HA-WT-MIP and HA-K228fs-MIP were determined after transient transfection in cultured HeLa cells. Confocal laser scanning images showed the distribution of HA-tagged MIP (red) and DAPI-stained nuclei (blue). HA-WT-MIP was detected mainly in the plasma membrane with less observed in the cytoplasm, but K228fs-MIP was largely localized in the cytoplasm. Scale bar: 5 μm

The reverse transcriptase quantitative PCR (RT-qPCR) analysis showed that mRNA transcription of K228fs-*MIP* was lower than that of WT-*MIP* (*T-TEST*, two-sided, *P = 0.0052, error bars represent the standard deviation, SD; Fig. 2a). Western blot analysis indicated that the p.K228fs mutation reduced MIP protein expression (Fig. 2b); β-actin expression was used as the control.

#### Localization of HA-WT-MIP and HA-K228fs-MIP in cultured cells

By confocal laser scanning, the localization of WT-MIP and K228fs-MIP in HeLa cells was detected individually after transient transfection. WT-MIP was mainly observed at the plasma membrane, which agrees with the distribution of a membrane protein in normal cells, whereas K228fs-MIP was primarily in the cytoplasm of HeLa cells (Fig. 2c). These images suggest that the mutant protein was trapped in the cytoplasm.

## Discussion

In this study, we investigated the genetic and functional defects of punctate cortical opacity cataracts in a four-generation Chinese family. WES and genealogical verification revealed a novel frameshift mutation of c.682_683delAA in the *MIP* gene. Using bioinformatics analysis, we speculated that the mutation may cause a substitution of 3-amino acids and a 33-amino-acid deletion from amino-acid 231 to the C-terminal by reason of a stop gain at amino-acid 231 of MIP, which may produce an incomplete C-terminus. Conservative analysis, physical/chemical parameters, and stability prediction suggests that the mutant loses most of the conserved MIP C-terminal structures, thereby affecting MIP stability and hydrophilicity. We suggest that this mutation caused the cataract phenotype in this family. To date, approximately 22 mutations have been identified in the *MIP* gene that are associated with a cataract phenotype: 16 missense/nonsense mutations [9–11, 13–22], 2 splicing variants [23, 24], 2 small deletions [25, 26] and 2 small insertions [12, 27].

As shown in supplement-Fig. 2, MIP plays a normal physiological role as an intrinsic membrane protein. It forms a water channel in the lens cells of the eye and is required for lens transparency and accommodation. The aquaporins perform normal physiological functions as a tetramer and normally are observed in the plasma membrane [28, 29]. MIP has a significant function in the maintenance of the permeability of water in the lens [30]. Mutations in the *MIP* gene can lead to variant cataract phenotypes. The clinical phenotype of this family was similar to those reported in previous studies [20, 26].

In this study, we detected a decrease in mRNA transcription in mutants using RT-qPCR and lower expression of K228fs-MIP protein than of WT-MIP protein by western blotting after the HA-WT-*MIP* and HA-K228fs-*MIP* vectors were individually transfected into HeLa cells. Several other mutations, such as p.G165D [9], p.Y219X [10], p.G212R [11], p.E134G [13], p.D150H [22], and p.G213Vfs [26], have been functionally characterized in vitro. All the above results indicated that the mutation generated unstable or variant proteins. The frameshift and truncation mutation caused by c.682_683delAA could cause RNA instability by nonsense-mediated mRNA decay, affecting post-translational modification or accelerating protein degradation. Moreover, confocal laser scanning immunofluorescence images indicated that WT-MIP protein was observed at the plasma membrane, which is consistent with the distribution of membrane proteins in cells. However, most of the K228fs-MIP protein was observed in the cytoplasm, which is similar to previously reported studies [9–11, 22]. The site at MIP Ser235 is required for proper MIP translocation to the plasma membrane by PKC-dependent phosphorylation [31]. A 28-amino-acid residue deletion from amino-acid 235–263 in *MIP* results in the retention of the mutant protein in the cytoplasm [32]. The region of the C-terminal from amino acids 223–234 in MIP is critical and may encode sorting signals for protein transport from the cytoplasm to the plasma membrane [8]. We speculate that the p.K228fs mutation prevents MIP protein transport by deleting important signal sites, such as Ser235. Therefore, these mutant proteins are inevitably trapped in the cytoplasm, which consequently reduces the formation of available water channels in the plasma membrane and affects lens fiber cell permeability and reduces transparency.

Of note, in this family the age of onset in male patients, which was within 1 year of age, was earlier than in female patients, who exhibited onset in adulthood; female patients also usually showed fewer symptoms. We are unsure whether this is chance or whether a second mutation influencing the age of onset is also segregating in this pedigree. Although we analyzed WES data from several patients, we did not identify a possible “second mutation” that would account for the phenotypic differences. Therefore, the c.682_683delAA mutation might not only impact protein expression level and localization but could might impact interactions with other undiscovered molecules. Hence, the specific causes of phenotypic heterogeneity in this family require further research.

In summary, we reported autosomal dominant congenital cataracts caused by a novel p.K228fs frameshift mutation in the *MIP* gene in a four-generation Chinese family. This study presented evidence that the mRNA and protein levels of K228fs-MIP were reduced and that the mutant protein was trapped in the cytoplasm. Our data extend the spectrum of known *MIP* gene mutations. In addition, we observed for the first time the phenomenon that the c.682_683delAA mutation in the *MIP* gene may result in age of onset phenotypic heterogeneity by gender; only a single family was assessed, and it is unclear if this is chance or via an unclear mechanism. Our study also presented evidence that WES is an effective method to assess a heterogeneous group of monogenic disorders, such as congenital cataracts.

## Summary

### What was known before

- Congenital cataract is a highly clinically and genetically heterogeneous disorder that caused by the opacity of the crystalline lens.
- About 36 nonsyndromic congenital cataract-related genes have been identified.
- Twenty-two mutations in MIP gene that linked to congenital cataracts have been identified in human.

### What this study adds

- A novel c.682_683delAA mutation in MIP gene can cause autosomal dominant congenital cataracts.
- The mRNA and protein levels of mutant were reduced and inappropriate positioning was also happened.
- The c.682_683delAA mutation of MIP may cause phenotypic heterogeneity between male and femal.

## Electronic supplementary material

(DOCX 16 kb)

Supplementary Figures and Tables(DOCX 13 kb)

Supplemental Table 1(DOCX 15 kb)

Supplemental Table 2(DOCX 17 kb)

Supplemental Table 3(DOCX 15 kb)

Supplemental Figure 1(TIF 553 kb)

Supplemental Figure 2(TIF 2129 kb)

Supplemental Figure 3(TIF 1402 kb)

Supplemental Figure 4(TIF 540 kb)
