# Supplementary material for: Identification of a novel MIP frameshift mutation associated with congenital cataract in a Chinese family by whole-exome sequencing and functional analysis
Source: Eye (Lond). 2018 Apr 26;32(8):1359–64. doi: 10.1038/s41433-018-0084-5 (PMC6085365; doi:10.1038/s41433-018-0084-5)
Supplement: Supplementary file 1 — (DOCX 16 kb) [file 41433_2018_84_MOESM1_ESM.docx]

**Supplement-Materials and methods**

***Supplement-Bioinformatics analysis (new added)***

The structures of protein were analyzed by UniProtKB (http://www.uniprot.org/). The amino acid conservation was analysed with mutation t@sting (http://www.mutationtaster.org/) and PolyPhen-2 (http://genetics.bwh.harvard.edu/pph2/). The physical and chemical parameters of the WT and K228fs proteins were assessed by ProtParam (http://web.expasy.org/protparam/). The stability of WT and K228fs were predicted with the BEST/COREX server (http://best.bio.jhu.edu/BEST/index.php).

**Supplement-Results**

***Supplement-Exome sequencing results***

By exome sequencing, only three rare variants were identified in the three samples (Supplement-Table 2). Two of these three rare variants were in the *RAB3GAP2* gene, which has been reported to be an autosomal recessive gene that mainly causes Warburg micro syndrome 2 or Martsolf syndrome ^33, 34^, and in the *ESCO2* gene, which has been reported to be an autosomal recessive allele that causes Roberts syndrome ^35^. These mutations have not noticeably affected the patients, as the disease in this family exhibited a dominant pattern and did not show other syndrome symptoms. Variants in the *GLYCTK, BCR, FANCD2* and *HYDIN* genes were assessed as pathogenic or likely pathogenic variants according to the standards and guidelines for the interpretation of sequence variants of the American College of Medical Genetics and Genomics (ACMG). However, none of the reported phenotypes conformed to the patient phenotype and are reported to show autosomal recessive inheritance. The exception is the *BCR* gene, which causes leukaemia by somatic mutation and does not conform to this family, which exhibited autosomal dominant inheritance.

***Supplement-Bioinformatics analysis of K228fs at the protein level (new added)***

Based on the description of UniProtKB-P30301(http://www.uniprot.org/uniprot/P30301), a schematic diagram of MIP was generated that shows the presumed membrane topology (Supplement-Fig. 2). The mutant is predicted to be truncated at amino acid 230 at the C-terminus. Conservative analysis suggested that the amino acid after the truncation showed high conservation (Supplement-Fig. 3). In addition, truncated MIP showed a higher instability index and grand average of hydropathicity (Supplement-Table 3). The stability constant (log Kf) at the per residue level shows that the mutant favoured unfolded states at the MIP C-terminus (Supplement-Fig. 4).
