# Supplementary material for: Identification of a novel MIP frameshift mutation associated with congenital cataract in a Chinese family by whole-exome sequencing and functional analysis
Source: Eye (Lond). 2018 Apr 26;32(8):1359–64. doi: 10.1038/s41433-018-0084-5 (PMC6085365; doi:10.1038/s41433-018-0084-5)
Supplement: Supplementary file 2 — Supplementary Figures and Tables(DOCX 13 kb) [file 41433_2018_84_MOESM2_ESM.docx]

**Titles and Legends to Supplementary Figures and Tables**

**Supplement-Figure 1**. Analysis process of whole exome sequencing data. QC: Quality Control;

PTM: Protein Function affected Mutation; MAF: Minor Allele Frequency.

**Supplement-Table 1** Primers sequence for Sanger sequencing

**Supplement-Table 2** Rare variants of congenital cataract-causing genes and the pathogenic or likely pathogenic genes predicted according to ACMG. *PopFreqMax means the Maximum Allele Frequency in the 1000G, ESP6500 and ExAC database. a# means patient phenotypes did not conform to the reported phenotype. b## means patient phenotypes conformed or partially conformed to the reported phenotypes.

**Supplement-Table 3** The physical and chemical parameters of WT and K228fs, analysed with ProtParam.

**Supplement-Figure** **2**. A schematic diagram of MIP shows the presumed membrane topology (based on the description of UniProtKB-P30301, http://www.uniprot.org/uniprot/P30301). MIP performs normal physiological functions as a tetramer and localizes to the plasma membrane. It has six transmembrane domains (H1-H6), two intracellular loops (B and D), three extracellular loops (A, C and E), and N- and C-terminal intracellular domains. The depicted portion (red circles) illustrates these transformational proteins, and the marked region (yellow circle) illustrates premature truncation of the protein. Amino acids after residue 220 are located in the cytoplasm.

**Supplement-Figure 3.** Multiple sequence alignment of MIP in different species indicates that the regions after the truncation were highly conserved.

**Supplement-Figure 4.** Residue-specific stability constant for each MIP residue as predicted by the BEST/COREX server. The C-terminal domain of truncated MIP exhibited the unfolded state. The stability constant (log Kf) at the per residue level is the ratio of the summed probability of the residue in folded and unfolded conformations.
