# Supplementary material for: Identification of a novel MIP frameshift mutation associated with congenital cataract in a Chinese family by whole-exome sequencing and functional analysis
Source: Eye (Lond). 2018 Apr 26;32(8):1359–64. doi: 10.1038/s41433-018-0084-5 (PMC6085365; doi:10.1038/s41433-018-0084-5)
Supplement: Supplementary file 3 — Supplemental Table 1(DOCX 15 kb) [file 41433_2018_84_MOESM3_ESM.docx]

Supplement-Table1 Primers sequences

| Primer | Sequence |
| --- | --- |
| Sense-1 | 5′-TGTGGGATAAAGGAGTAATTTGATGA-3′ |
| Anti-sense-1 | 5′-CTTCATCTAGGGGCTGGCTAAAC-3′ |
| Sense-2 | 5′-CGCGGATCCATGTGGGAACTGCGATCAGC-3′ |
| Anti-sense-2 | 5′-ACGCGTCGACCTACAGGGCCTGGGTGTTCAG-3′ |
| sense-3 | 5′-CGGCTcgAGTATTTCTGAGAGACTGTCTGTCCTCA-3′ |
| Anti-sense-3 | 5′-TCAGAAATACTcgAGCCGGGGGAAGAGAAGAAA-3′ |
| sense-4 | 5′-CATTCTCACTGGGAACTTCACTAAC-3′ |
| Anti-sense-4 | 5′- GCACCCTTGAGGACAGACAGT-3′ |
| sense-5 | 5′- GCACAGAGCCTCGCCTT-3′ |
| Anti-sense-5 | 5′- CCTTGCACATGCCGGAG-3′ |
