# Supplementary material for: Identification of a novel MIP frameshift mutation associated with congenital cataract in a Chinese family by whole-exome sequencing and functional analysis
Source: Eye (Lond). 2018 Apr 26;32(8):1359–64. doi: 10.1038/s41433-018-0084-5 (PMC6085365; doi:10.1038/s41433-018-0084-5)
Supplement: Supplementary file 4 — Supplemental Table 2(DOCX 17 kb) [file 41433_2018_84_MOESM4_ESM.docx]

Table1. Rare variants of congenital cataract causing genes and the pathogenic or likely pathogenic genes that were predicted according to ACMG.

| Location | Gene | Exon | Genotype | Transcript | DNA Change | Residue Change | Function | PopFreqMax* | Evidence | Level | phenotype |
| --- | --- | --- | --- | --- | --- | --- | --- | --- | --- | --- | --- |
| chr3 | *GLYCTK* | exon2 | het | NM_001144951.1 | c.C37T | p.R13X | stop gain | 0 | pvs1 pm2 pp3 | Pathogenic | a# |
| chr22 | *BCR* | exon19 | het | NM_004327.3 | c.3274_3275insCCGG | p.S1092fs | frameshift insertion | 0 | pvs1 pm2 | Likely pathogenic | a# |
| chr3 | *FANCD2* | exon15 | het | NM_001018115.1 | c.1278_1278del | p.L426fs | frameshift deletion | 0 | pvs1 pm2 | Likely pathogenic | a# |
| chr16 | *HYDIN* | exon69 | het | NM_001270974.1 | c.11712delT | p.I3904fs | frameshift deletion | 0 | pvs1 pm2 | Likely pathogenic | a# |
| chr12 | *MIP* | exon4 | het | NM_012064.3 | c.682_683del | p.K228fs | frameshift deletion | 0 | pvs1 pm2 | Likely pathogenic | b## |
| chr1 | *RAB3GAP2* | exon27 | het | NM_012414.3 | c.A3143G | p.H1048R | nonsynonymous SNV | 0.004 | pm2 | Uncertain | b## |
| chr8 | *ESCO2* | UTR3 | het | NM_001017420.2 | c.*111_*112insAC | - | - | 0 | pm2 | Uncertain | b## |

*PopFreqMax means the maximum allele frequency in these databases of 1000G, ESP6500 and ExAC. a# means patients’ phenotypes didn’t conform to the reported phenotype. b## means patients’ phenotypes were conformed or partially conformed to the reported phenotypes.
