# Supplementary material for: Identification of a novel MIP frameshift mutation associated with congenital cataract in a Chinese family by whole-exome sequencing and functional analysis
Source: Eye (Lond). 2018 Apr 26;32(8):1359–64. doi: 10.1038/s41433-018-0084-5 (PMC6085365; doi:10.1038/s41433-018-0084-5)
Supplement: Supplementary file 5 — Supplemental Table 3(DOCX 15 kb) [file 41433_2018_84_MOESM5_ESM.docx]

Table 2 The physical and chemical parameters of WT and K228fs were analyzed by ProtParam

| Protein characteristics | WT | K228fs |
| --- | --- | --- |
| Number of amino acids | 263 | 230 |
| Molecular weight | 28121.75 | 24786.98 |
| Theoretical PI | 8.62 | 8.66 |
| Instablity index | 34.04 | 37.94 |
| Aliphatic index | 105.74 | 106.52 |
| Grand average of hydropathicity | 0.613 | 0.771 |
|  | | |
